# Supplementary material for: MolPhase, an advanced prediction algorithm for protein phase separation
Source: EMBO J. 2024 Apr 2;43(9):10. doi: 10.1038/s44318-024-00090-9 (PMC11065880; doi:10.1038/s44318-024-00090-9)
Supplement: Supplementary file 1 — Table EV1 [file 44318_2024_90_MOESM1_ESM.docx]

**Table EV1. Definitions and Reference Tools for Features Used in MolPhase Predictions.**

| **Feature** | **Definition** | **Tools / Ref** |
| --- | --- | --- |
| IDR percentage | Assign disorder state to a residue when the meta-predictor MobiDB-lite reaches the agreement of at least 62.5% (5 out of 8 predictors).  IDR percentage = Length(IDR sequence) / Length (total sequence)  For the length of IDR sequence, only IDR longer than 5 consecutive disordered residues would be counted. | MobiDB-lite 3.0 (Necci *et al*, 2020) |
| LCR percentage | Extract the LCRs by SEG  LCR percentage = Length(LCR) / Length (total sequence) | SEG algorithm (trigger window length W=25, trigger complexity K1=3.0, extension complexity K2=3.3) (Wootton & Federhen, 1993) |
| PLD | Log likelihood of PLD = The max sum of PLAAC log-likelihood ratios (base 4) in window of size 60 | PLAAC (Lancaster *et al*, 2014) |
| Shannon Entropy | $H\left( X \right)=-\sum_{i=1}^{N=20} p_{i}{log}_{2}p_{i}$  where, i is the amino acid in the sequence (i = A, C, D, …, Y) and X is any protein / peptide sequence. | SciPy (Virtanen *et al*, 2020) |
| FCR | FCR = $f_{+}+f_{-}$  where $f_{+}$means portal of positive charge residues, $f_{-}$ means portal of negative residues, window size set as 5 residues. | Localcider (Holehouse *et al*, 2017) |
| NCPR | NCPR = $f_{+}-f_{-}$  where $f_{+}$means portal of positive charge residues, $f_{-}$ means portal of negative residues, window size set as 5 residues. | Localcider (Holehouse *et al.*, 2017) |
| Kappa | The mixing pattern of charged residues | Localcider (Das & Pappu, 2013; Holehouse *et al.*, 2017) |
| Omega | The mixing pattern of charged/proline residues and all other residues | Localcider (Holehouse *et al.*, 2017; Martin *et al*, 2016) |
| Polyproline II propensity | The polyproline II conformation surrounding intrinsically disordered phosphorylation sites | Localcider (Austin Elam *et al*, 2013; Holehouse *et al.*, 2017) |
| Pi-Pi interaction | Pi-Pi interactions frequency | PScore (Vernon *et al*, 2018) |
| Mean hydropathy | Normalized Kyte-Doolittle hydrophobicity scale, ranging from 0 to 1 | Localcider (Holehouse *et al.*, 2017; Kyte & Doolittle, 1982) |

**References**

Austin Elam W, Schrank TP, Campagnolo AJ, Hilser VJ (2013) Evolutionary conservation of the polyproline II conformation surrounding intrinsically disordered phosphorylation sites. *Protein Science* 22: 405-417

Das RK, Pappu RV (2013) Conformations of intrinsically disordered proteins are influenced by linear sequence distributions of oppositely charged residues. *Proceedings of the National Academy of Sciences* 110: 13392-13397

Holehouse AS, Das RK, Ahad JN, Richardson MO, Pappu RV (2017) CIDER: resources to analyze sequence-ensemble relationships of intrinsically disordered proteins. *Biophysical journal* 112: 16-21

Kyte J, Doolittle RF (1982) A simple method for displaying the hydropathic character of a protein. *Journal of molecular biology* 157: 105-132

Lancaster AK, Nutter-Upham A, Lindquist S, King OD (2014) PLAAC: a web and command-line application to identify proteins with prion-like amino acid composition. *Bioinformatics* 30: 2501-2502

Martin EW, Holehouse AS, Grace CR, Hughes A, Pappu RV, Mittag T (2016) Sequence determinants of the conformational properties of an intrinsically disordered protein prior to and upon multisite phosphorylation. *Journal of the American Chemical Society* 138: 15323-15335

Necci M, Piovesan D, Clementel D, Dosztányi Z, Tosatto SC (2020) MobiDB-lite 3.0: fast consensus annotation of intrinsic disorder flavors in proteins. *Bioinformatics* 36: 5533-5534

Vernon RM, Chong PA, Tsang B, Kim TH, Bah A, Farber P, Lin H, Forman-Kay JD (2018) Pi-Pi contacts are an overlooked protein feature relevant to phase separation. *elife* 7: e31486

Virtanen P, Gommers R, Oliphant TE, Haberland M, Reddy T, Cournapeau D, Burovski E, Peterson P, Weckesser W, Bright J (2020) SciPy 1.0: fundamental algorithms for scientific computing in Python. *Nature methods* 17: 261-272

Wootton JC, Federhen S (1993) Statistics of local complexity in amino acid sequences and sequence databases. *Computers & chemistry* 17: 149-163
