# Supplementary material for: MolPhase, an advanced prediction algorithm for protein phase separation
Source: EMBO J. 2024 Apr 2;43(9):10. doi: 10.1038/s44318-024-00090-9 (PMC11065880; doi:10.1038/s44318-024-00090-9)
Supplement: Supplementary file 2 — Table EV2 [file 44318_2024_90_MOESM2_ESM.docx]

**Table EV2.** The classification of 20 essential amino acids based on the propensities of residues.

| **Group** | **Amino Acid** | **Definition** |
| --- | --- | --- |
| Polar residues | Serine (S), Glutamine (Q), Asparagine (N), Glycine (G), Cysteine (C), Threonine (T), Proline (P) | Amino acids that are with polar uncharged side chains. |
| Hydrophobic residues | Alanine (A), Valine (V), Leucine (L), Isoleucine (I), Methionine (M), Phenylalanine (F) | Amino acids with side chains that do not like to reside in an aqueous (i.e., water) environment. |
| Aromatic residues | Tryptophan (W), Tyrosine (Y), Phenylalanine (F) | Amino acids include an aromatic ring. |
| Cationic residues | Lysine (K), Arginine (R), Histidine (H) | Amino acids that are positively charged at pH = 7.4. |
| Anionic residues | Aspartic acid (D), Glutamic acid (E) | Amino acids that are negatively charged at pH = 7.4. |
| Expanding residues | Aspartic acid (D), Glutamic acid (E), Lysine (K), Arginine (R), Proline (P) | Amino acids are predicted to contribute to chain expansion. |
| Disorder promoting residues | Threonine (T), Alanine (A), Glycine (G), Arginine (R), Aspartic acid (D), Histidine (H), Glutamine (Q), Lysine (K), Serine (S), Glutamic acid (E), Proline (P) | Amino acids which are predicted to be disorder promoting (Campen *et al*, 2008). |

**References**

Campen A, Williams RM, Brown CJ, Meng J, Uversky VN, Dunker AK (2008) TOP-IDP-scale: a new amino acid scale measuring propensity for intrinsic disorder. *Protein and peptide letters* 15: 956-963
