# Supplementary material for: MolPhase, an advanced prediction algorithm for protein phase separation
Source: EMBO J. 2024 Apr 2;43(9):10. doi: 10.1038/s44318-024-00090-9 (PMC11065880; doi:10.1038/s44318-024-00090-9)
Supplement: Supplementary file 3 — Table EV3 [file 44318_2024_90_MOESM3_ESM.docx]

**Table EV3. Primers used in this study.**

| **Primer name** | **Primer sequence** |
| --- | --- |
| HopA1_XVEarm-F | CTCTAGCCTCGAGGCGCGCCATGAACCCCATTCAGTCACGC |
| HopA1_XVEarm-R | TCGAGTGCGGCCGCAAGCTTTTTCGTGTTTCGAAGGGCC |
| HopAB2_XVEarm-F | TAGCCTCGAGGCGCGCCATGGCCGGTATAAATCGTGCTGG |
| HopAB2_XVEarm-R | TCTTCGCCCTTAGACACCATGTGGTGGTGGTGGTGGTG |
| HopS1_XVEarm-F | TAGCCTCGAGGCGCGCCATGAAAATATCCGGCTCCACATCG |
| HopS1_XVEarm-R | TCGAGTGCGGCCGCAAGCTTGACCTTCCCAAGCTCTGGATTT |
| XopQ_XVEarm-F | TAGCCTCGAGGCGCGCCATGGTGCCCGCAGGCGCTCAT |
| XopQ_XVEarm-R | TCGAGTGCGGCCGCAAGCTTTTGTTTCGTGGCAAGCGC |
| pER10_XVE_cds-F | AAGCTTGCGGCCGCACTC |
| pER10_XVE_cds-R | CATGGCGCGCCTCGAGGCTA |
| HopO1-2_XVEarm-F | TAGCCTCGAGGCGCGCCATGAATATCAGTCCTGTATCGGGTGC |
| HopO1-2_XVEarm-R | TCGAGTGCGGCCGCAAGCTTCTCGTCTGAATTATCTGCTATCTCGT |
| XopD2_XVEarm-F | CTCTAGCCTCGAGGCGCGCCATGGTGGAATCCCAAGACCC |
| XopD2_XVEarm-R | TCGAGTGCGGCCGCAAGCTTCGGCTGTATCGCGGTGGG |
| XopX2_XVEarm-F | CTAGCCTCGAGGCGCGCCATGCGCAGCCGGCGGCACAG |
| XopX2_XVEarm-R | TCGAGTGCGGCCGCAAGCTTGCCGGAGCGCGGCGGGTC |
| XopAH_XVEarm-F | TAGCCTCGAGGCGCGCCATGTGGTCTCAGCCCGTATGGAA |
| XopAH_XVEarm-R | TCGAGTGCGGCCGCAAGCTTAATTGGGGGGCGCTCAAA |
| XopAY_XVEarm-F | TAGCCTCGAGGCGCGCCATGGTGACTTCTGTGGCTCGTGAAGC |
| XopAY_XVEarm-R | TCGAGTGCGGCCGCAAGCTTCAAGAGCAGTTGCGTGACCA |
| XopAZ_XVEarm-F | TAGCCTCGAGGCGCGCCATGGAAATCACCCAGGGTCGCG |
| XopAZ_XVEarm-R | TCGAGTGCGGCCGCAAGCTTGGCGGCGATGTCGGTGAC |
| HopE1_XVEarm-F | TAGCCTCGAGGCGCGCCATGAATAGAGTTTCCGGTAGCTCGTCA |
| HopE1_XVEarm-R | TCGAGTGCGGCCGCAAGCTTGTCAATCACATGCGCTTGGC |
| HopQ1-2_XVEarm-F | CTCTAGCCTCGAGGCGCGCCATGAGCAGCCCAGCTTTGG |
| HopQ1-2_XVEarm-R | TCGAGTGCGGCCGCAAGCTTATCCGGGACTGCCGTCGA |
| XVE_to_pHGW-F | GCTACGCGTCTCGAGGTCGACTAGCCTCGAGGCGCGCCA |
| XVE_to_pHGW-R | TCCTCCAGAACCACCGAATTCGTGGTGGTGGTGGTGGTGC |
| pHGW_cds-F | GAATTCGGTGGTTCTGGAGGATC |
| pHGW_cds-R | GTCGACCTCGAGACGCGTAG |
| pSUMO_cds-F | TGAGATCCGGCTGCTAACAAA |
| pSUMO_cds-R | ACCCCCCGTTTGTTCCTG |
| HopA1_SUMOarm-F | ATCAGGAACAAACGGGGGGTATGAACCCCATTCAGTCACGC |
| HopA1_SUMOarm-R | TTGTTAGCAGCCGGATCTCATTTCGTGTTTCGAAGGGCC |
| XopQ_SUMOarm-F | ATCAGGAACAAACGGGGGGTATGGTGCCCGCAGGCGCT |
| XopQ_SUMOarm-R | TTGTTAGCAGCCGGATCTCATTGTTTCGTGGCAAGCGC |
| shcS1_XVEarm-F | TAAGAAGGAGATATACCATGAATGCGTTCGCAACCGGT |
| HopS1_6*Hisarm-R | CAGTGGTGGTGGTGGTGGTGGACCTTCCCAAGCTCTGGATTT |
