# Supplementary figures and images for: MolPhase, an advanced prediction algorithm for protein phase separation
Source: EMBO J. 2024 Apr 2;43(9):10. doi: 10.1038/s44318-024-00090-9 (PMC11065880; doi:10.1038/s44318-024-00090-9)

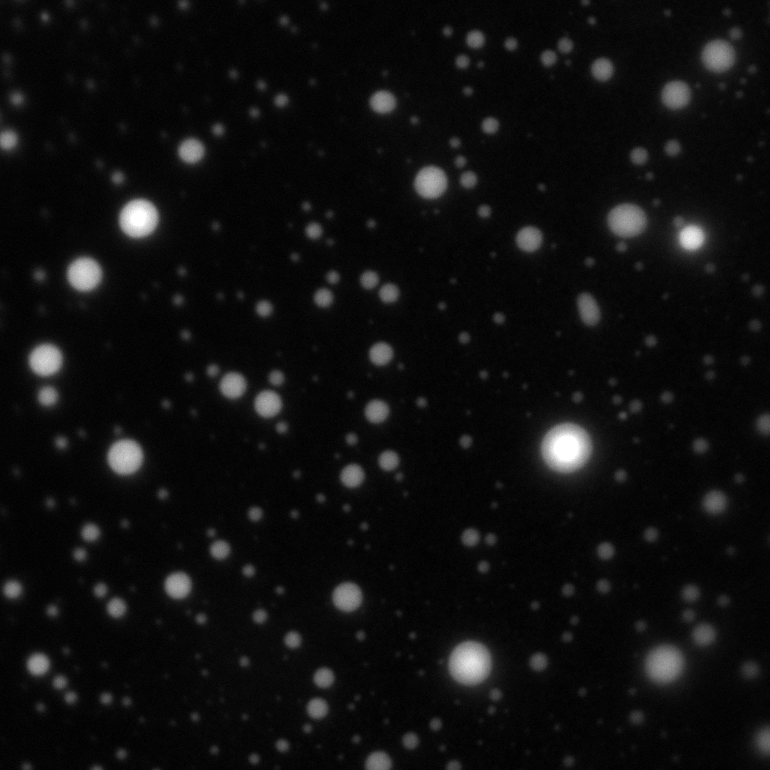

Supplement: Supplementary file 8 — Source data Fig. 4 [file 44318_2024_90_MOESM8_ESM.zip › Figure 4-Source Data Files/4A/Fig 4A-HopS1 Alexa 488.tif]

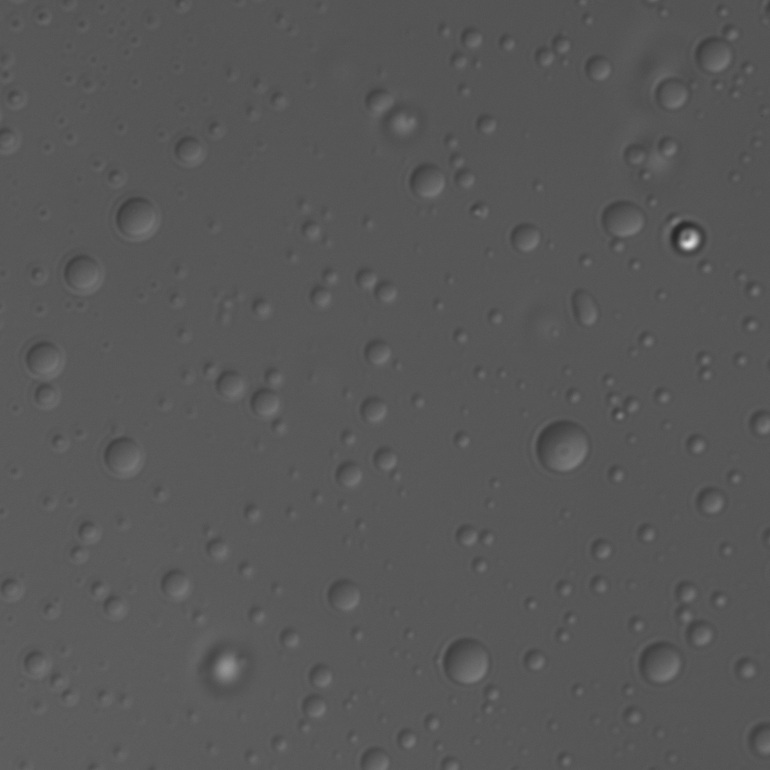

Supplement: Supplementary file 8 — Source data Fig. 4 [file 44318_2024_90_MOESM8_ESM.zip › Figure 4-Source Data Files/4A/Fig 4A-HopS1 DIC.tif]

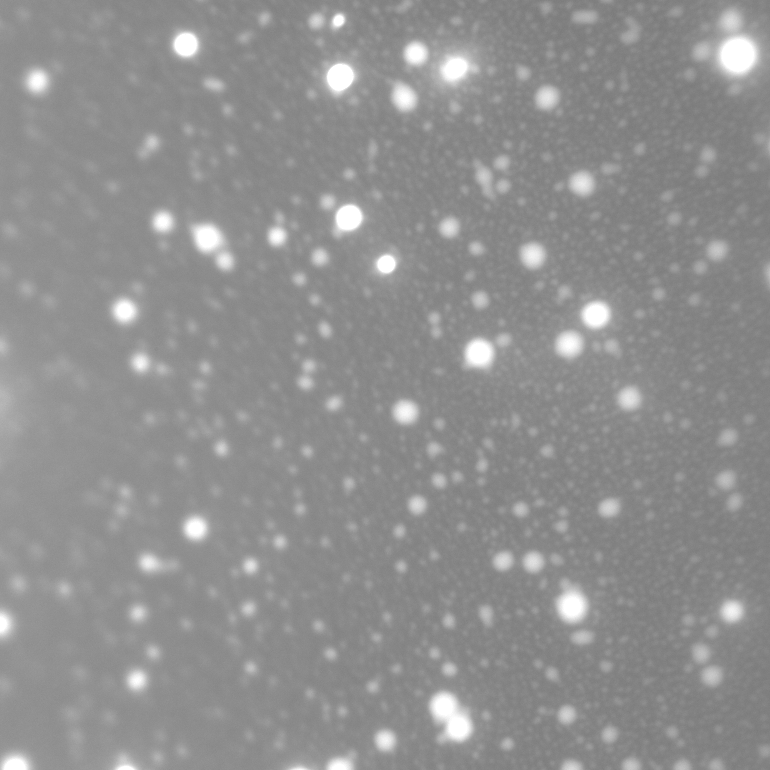

Supplement: Supplementary file 8 — Source data Fig. 4 [file 44318_2024_90_MOESM8_ESM.zip › Figure 4-Source Data Files/4B/Fig 4B-HopA1 Alexa 488.tif]

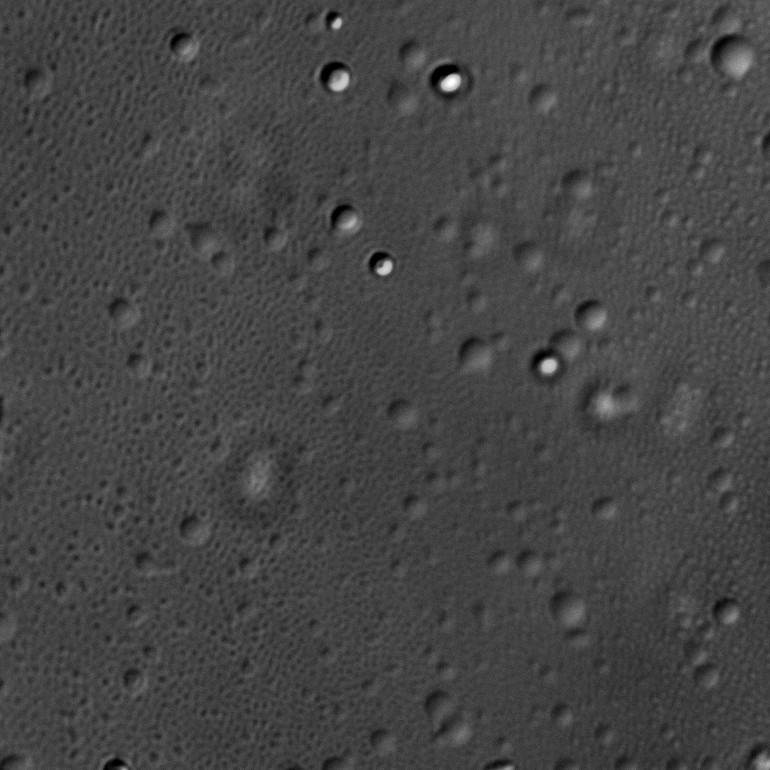

Supplement: Supplementary file 8 — Source data Fig. 4 [file 44318_2024_90_MOESM8_ESM.zip › Figure 4-Source Data Files/4B/Fig 4B-HopA1 DIC.tif]

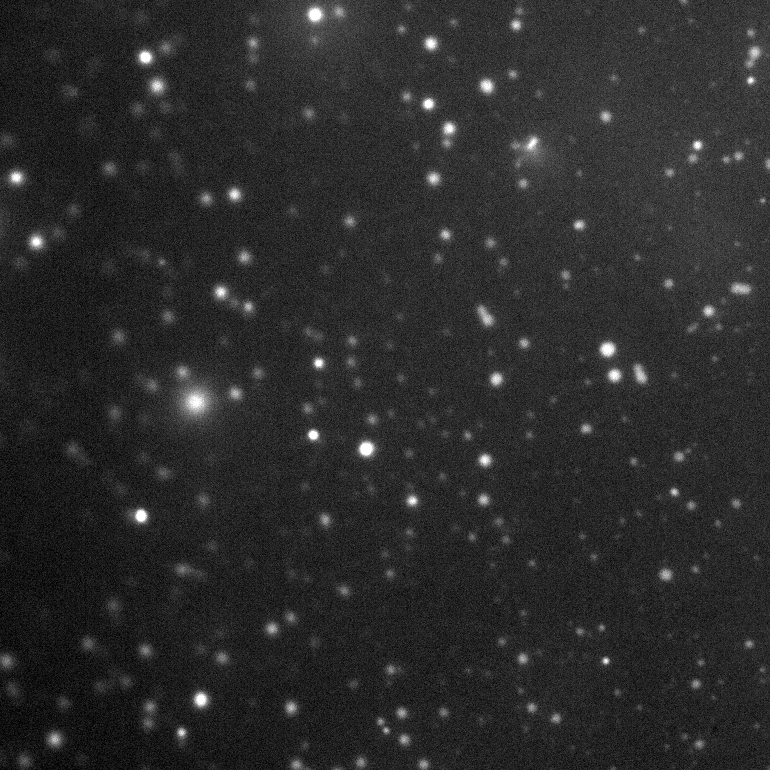

Supplement: Supplementary file 8 — Source data Fig. 4 [file 44318_2024_90_MOESM8_ESM.zip › Figure 4-Source Data Files/4C/Fig 4C-XopQ Alexa 488.tif]

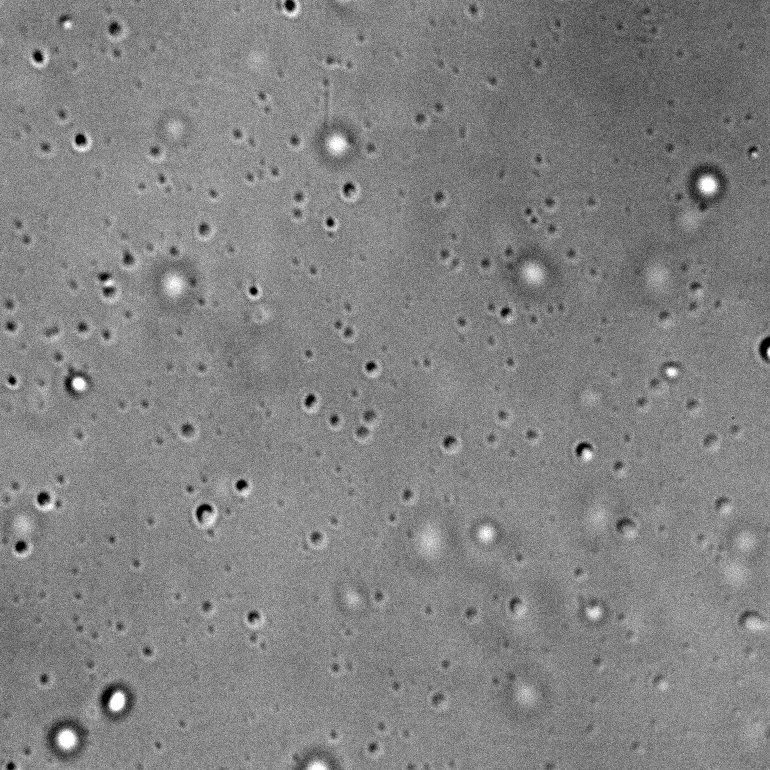

Supplement: Supplementary file 8 — Source data Fig. 4 [file 44318_2024_90_MOESM8_ESM.zip › Figure 4-Source Data Files/4C/Fig 4C-XopQ DIC.tif]

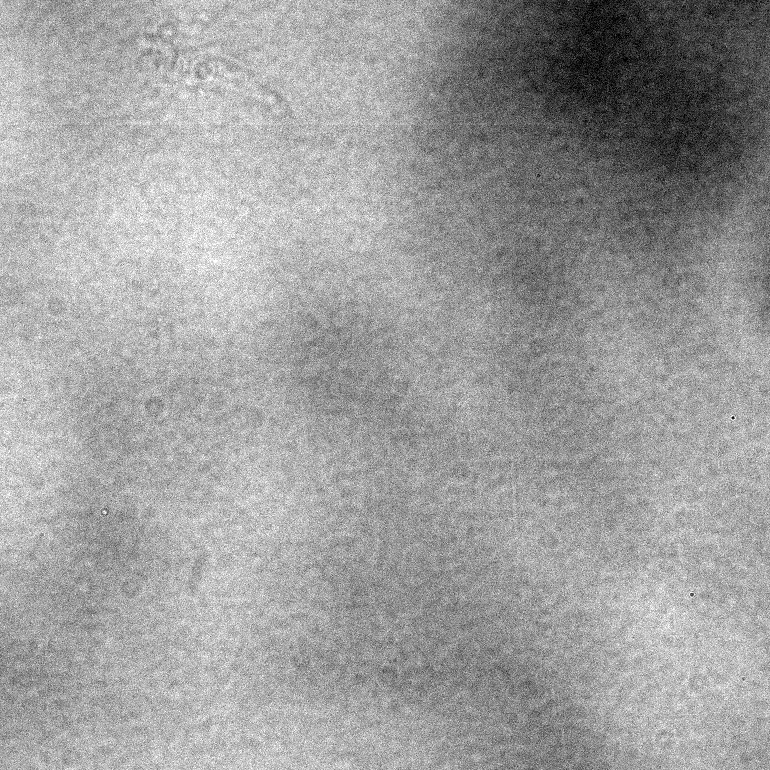

Supplement: Supplementary file 8 — Source data Fig. 4 [file 44318_2024_90_MOESM8_ESM.zip › Figure 4-Source Data Files/4D/Fig 4D-DIC.tif]

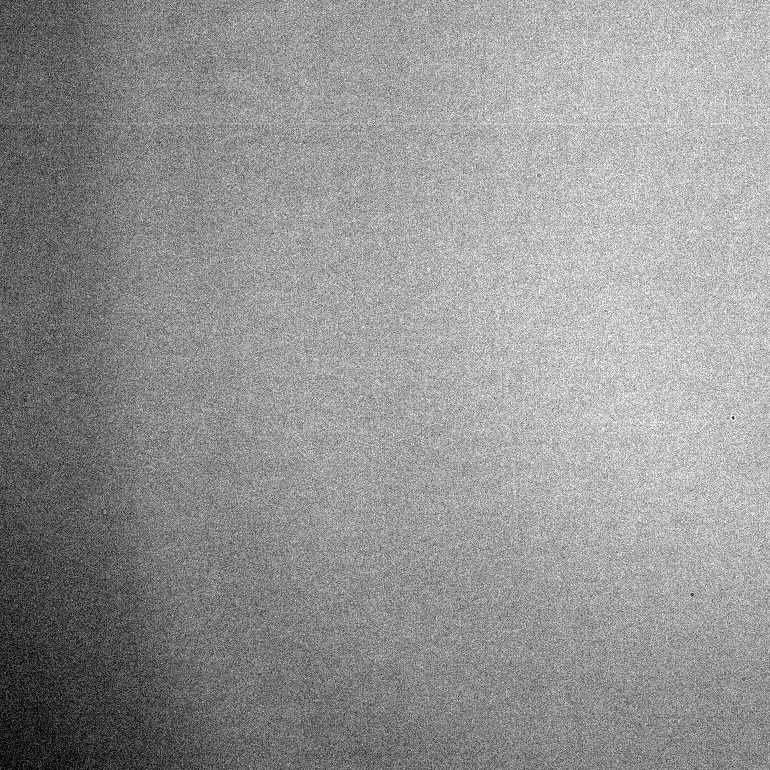

Supplement: Supplementary file 8 — Source data Fig. 4 [file 44318_2024_90_MOESM8_ESM.zip › Figure 4-Source Data Files/4D/Fig 4D-mRuby2.tif]

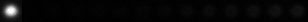

Supplement: Supplementary file 8 — Source data Fig. 4 [file 44318_2024_90_MOESM8_ESM.zip › Figure 4-Source Data Files/4E/Fig 4E-HopA1.tif]

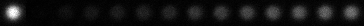

Supplement: Supplementary file 8 — Source data Fig. 4 [file 44318_2024_90_MOESM8_ESM.zip › Figure 4-Source Data Files/4E/Fig 4E-HopS1.tif]

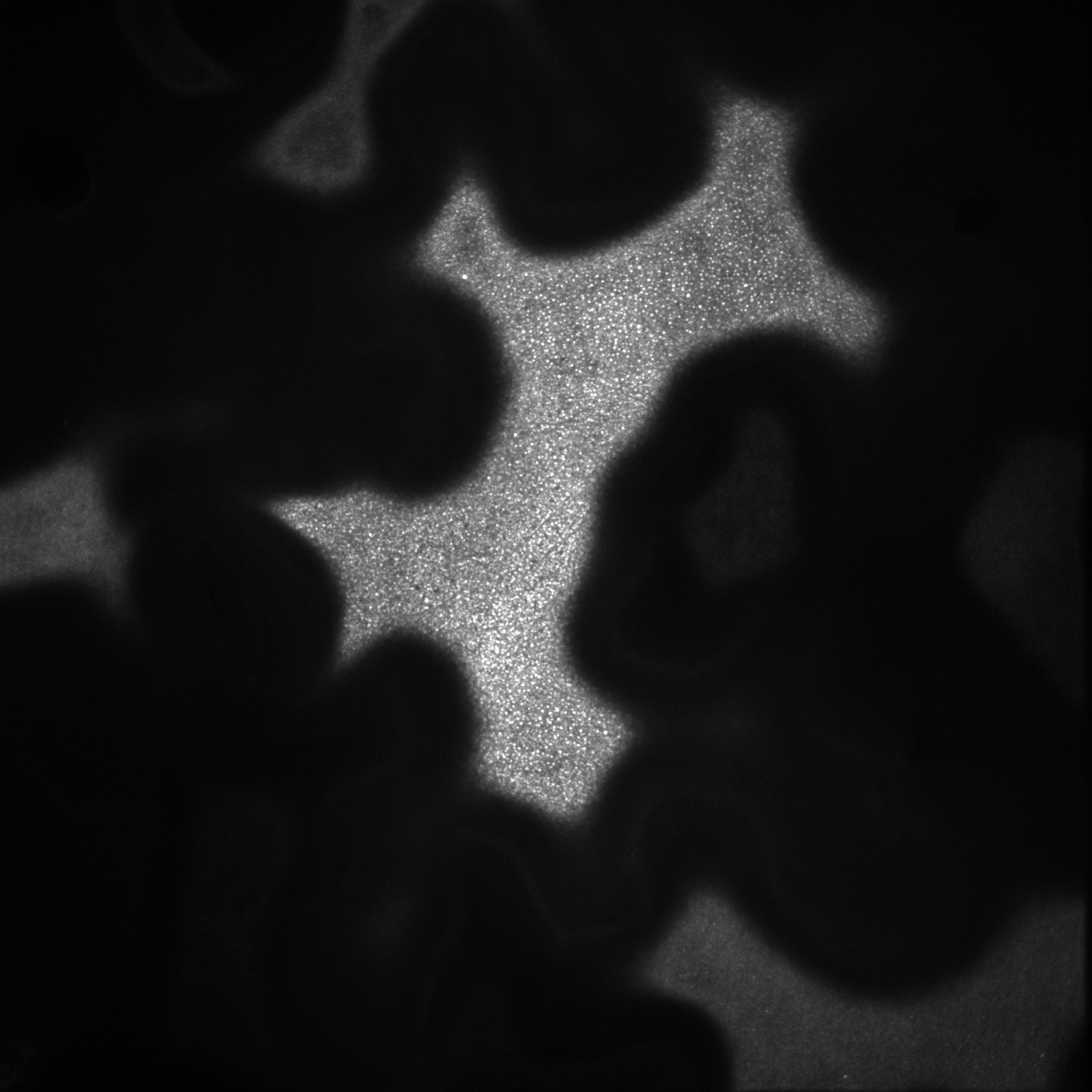

Supplement: Supplementary file 8 — Source data Fig. 4 [file 44318_2024_90_MOESM8_ESM.zip › Figure 4-Source Data Files/4G/Fig 4G-HopA1.tif]

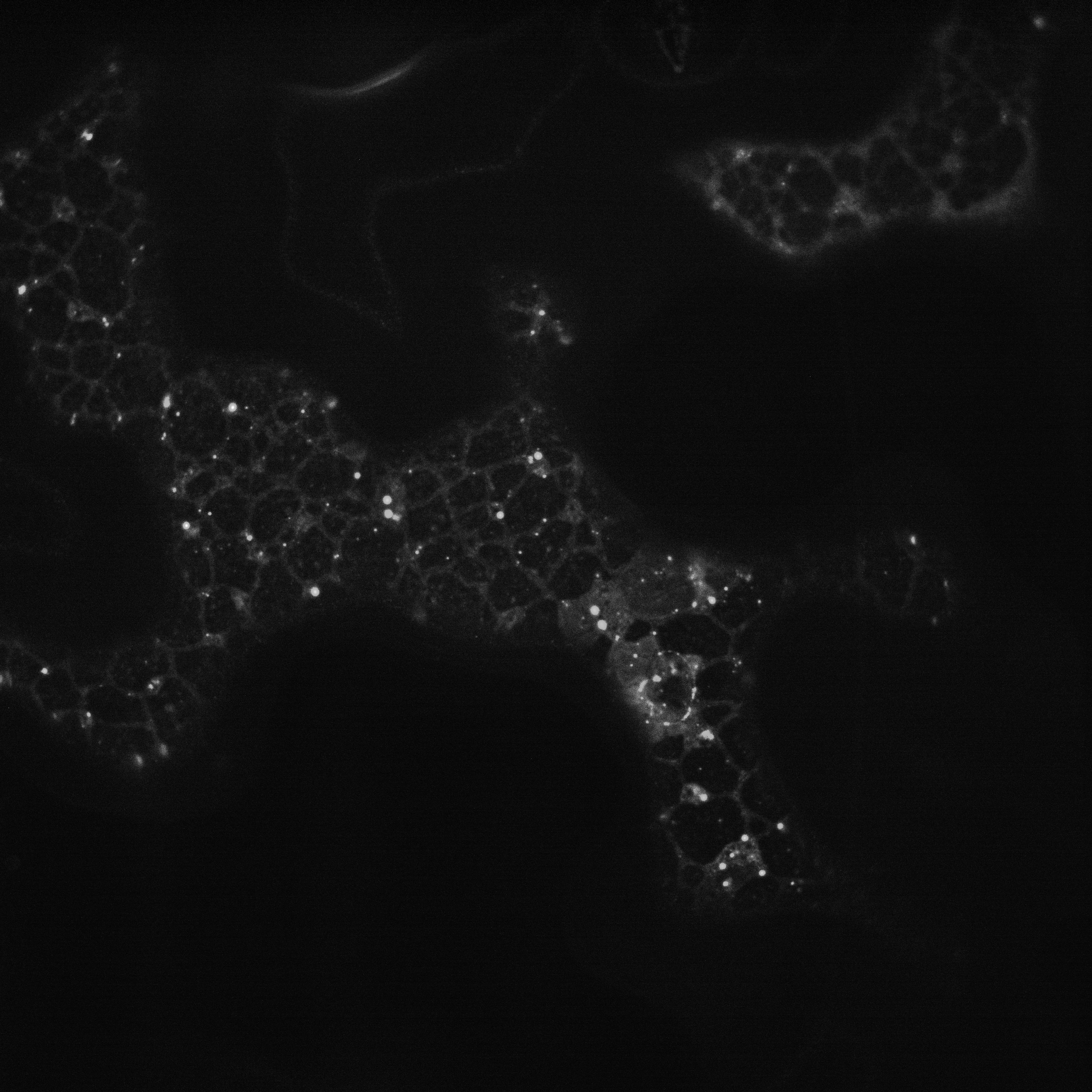

Supplement: Supplementary file 8 — Source data Fig. 4 [file 44318_2024_90_MOESM8_ESM.zip › Figure 4-Source Data Files/4G/Fig 4G-HopAB2.tif]

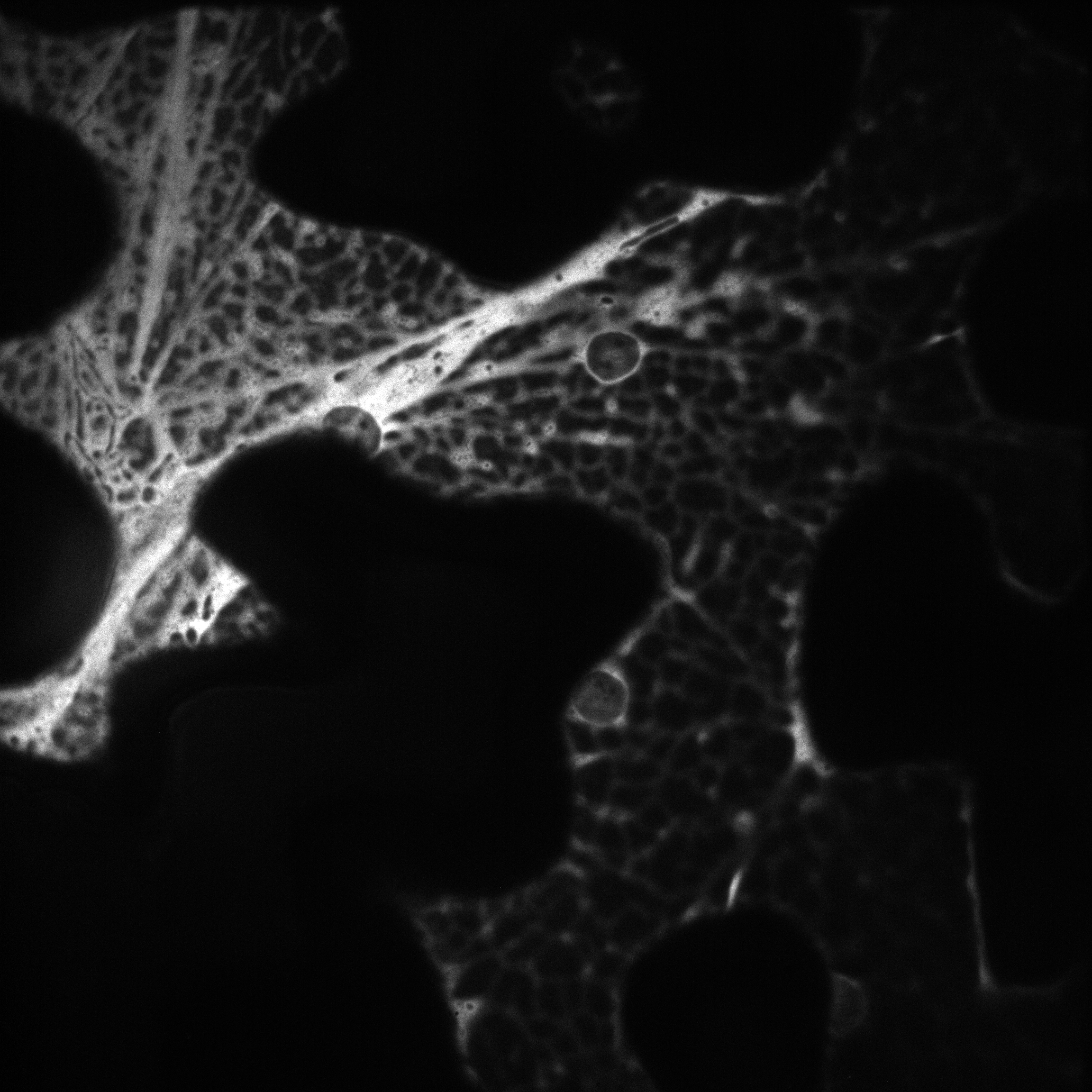

Supplement: Supplementary file 8 — Source data Fig. 4 [file 44318_2024_90_MOESM8_ESM.zip › Figure 4-Source Data Files/4G/Fig 4G-HopE1.tif]

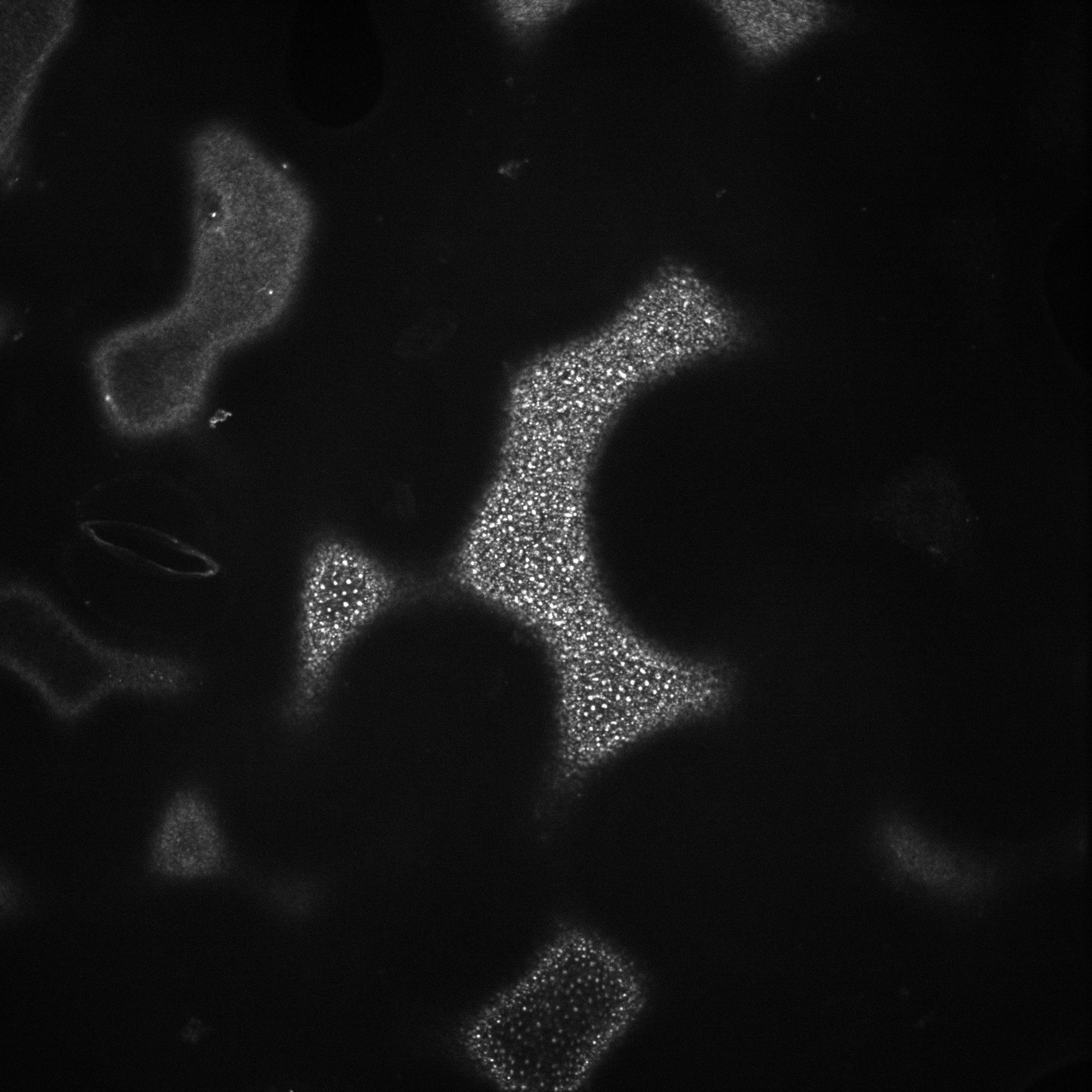

Supplement: Supplementary file 8 — Source data Fig. 4 [file 44318_2024_90_MOESM8_ESM.zip › Figure 4-Source Data Files/4G/Fig 4G-HopO1-2.tif]

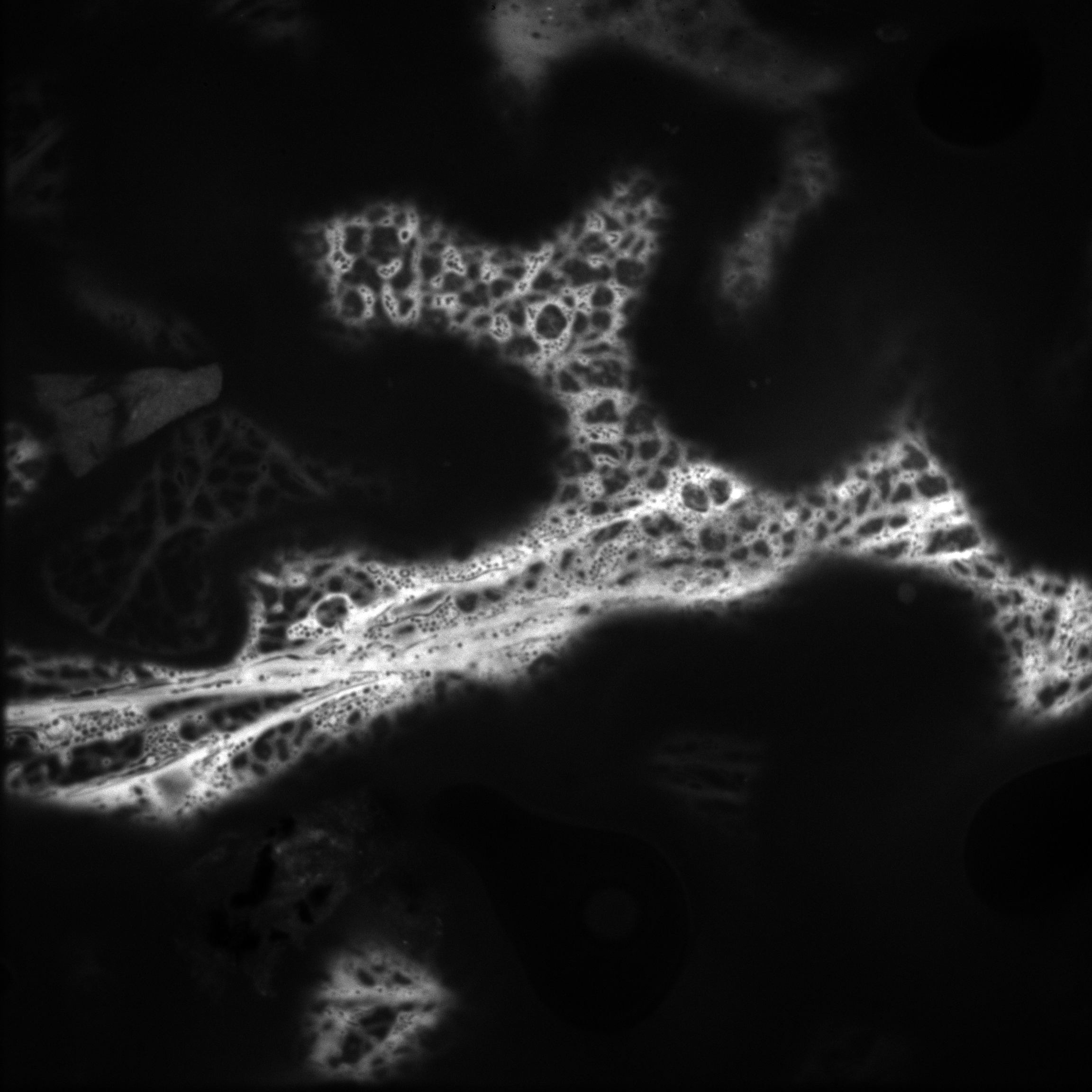

Supplement: Supplementary file 8 — Source data Fig. 4 [file 44318_2024_90_MOESM8_ESM.zip › Figure 4-Source Data Files/4G/Fig 4G-HopQ1-2.tif]

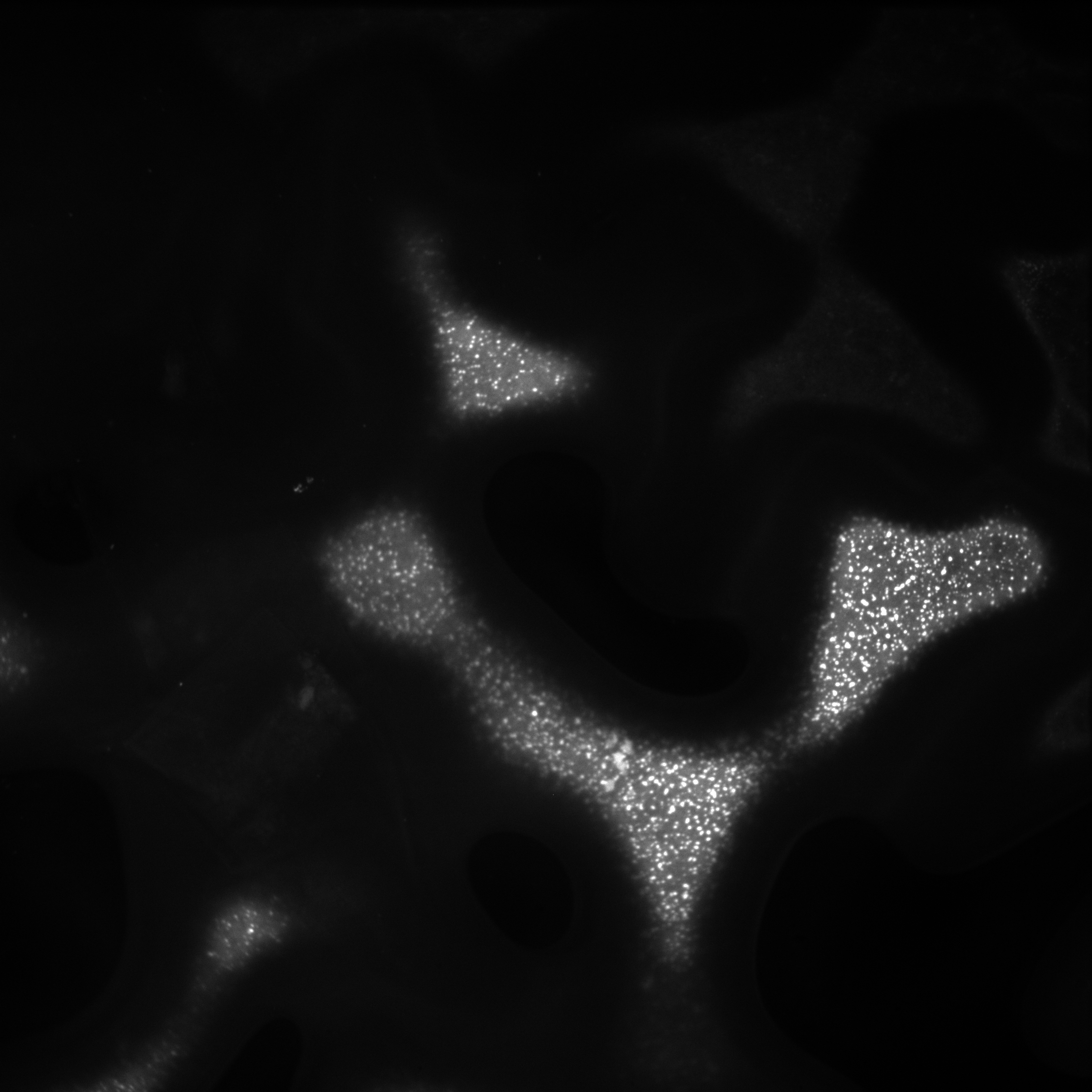

Supplement: Supplementary file 8 — Source data Fig. 4 [file 44318_2024_90_MOESM8_ESM.zip › Figure 4-Source Data Files/4G/Fig 4G-HopS1.tif]

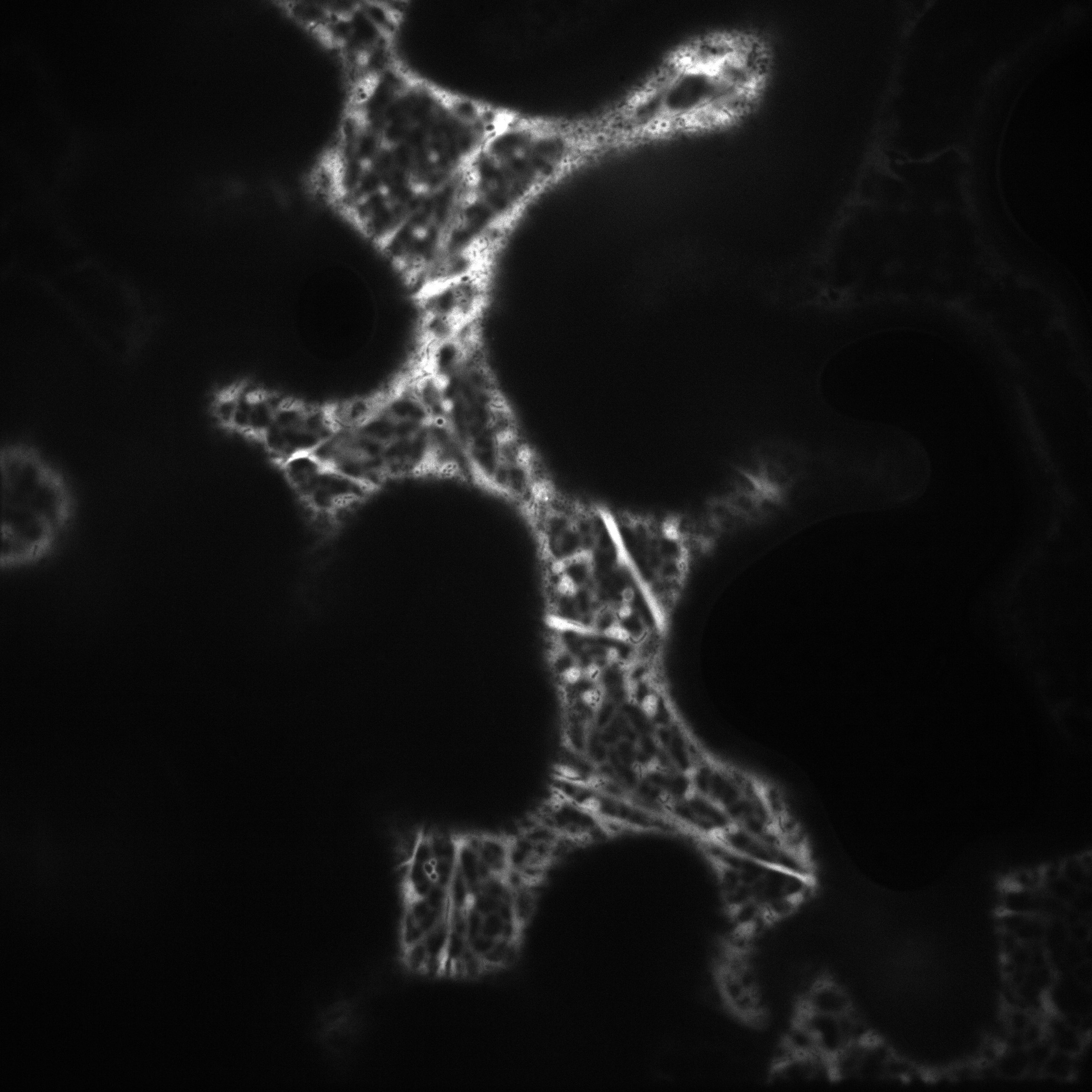

Supplement: Supplementary file 8 — Source data Fig. 4 [file 44318_2024_90_MOESM8_ESM.zip › Figure 4-Source Data Files/4G/Fig 4G-mRuby2.tif]

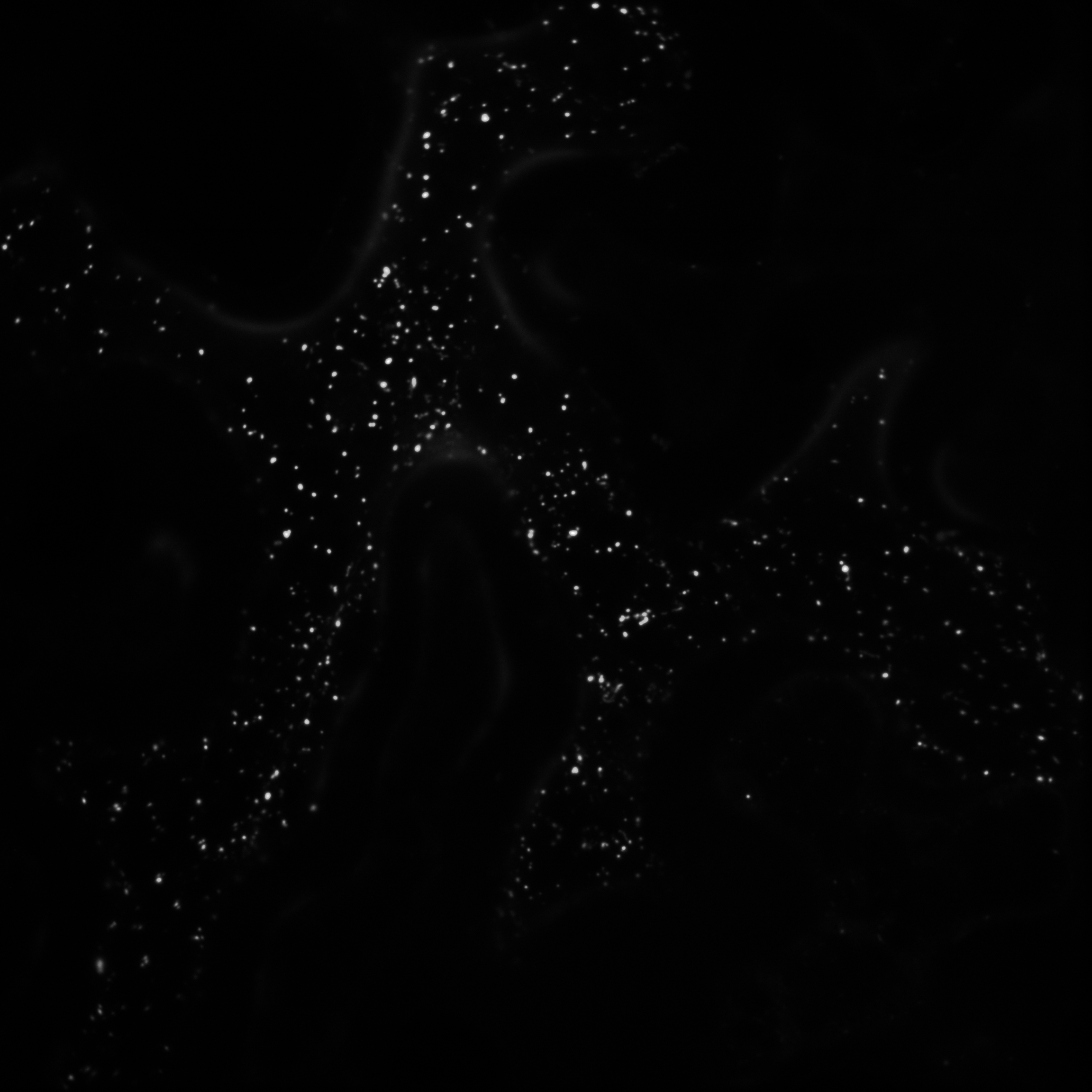

Supplement: Supplementary file 8 — Source data Fig. 4 [file 44318_2024_90_MOESM8_ESM.zip › Figure 4-Source Data Files/4G/Fig 4G-XopAH.tif]

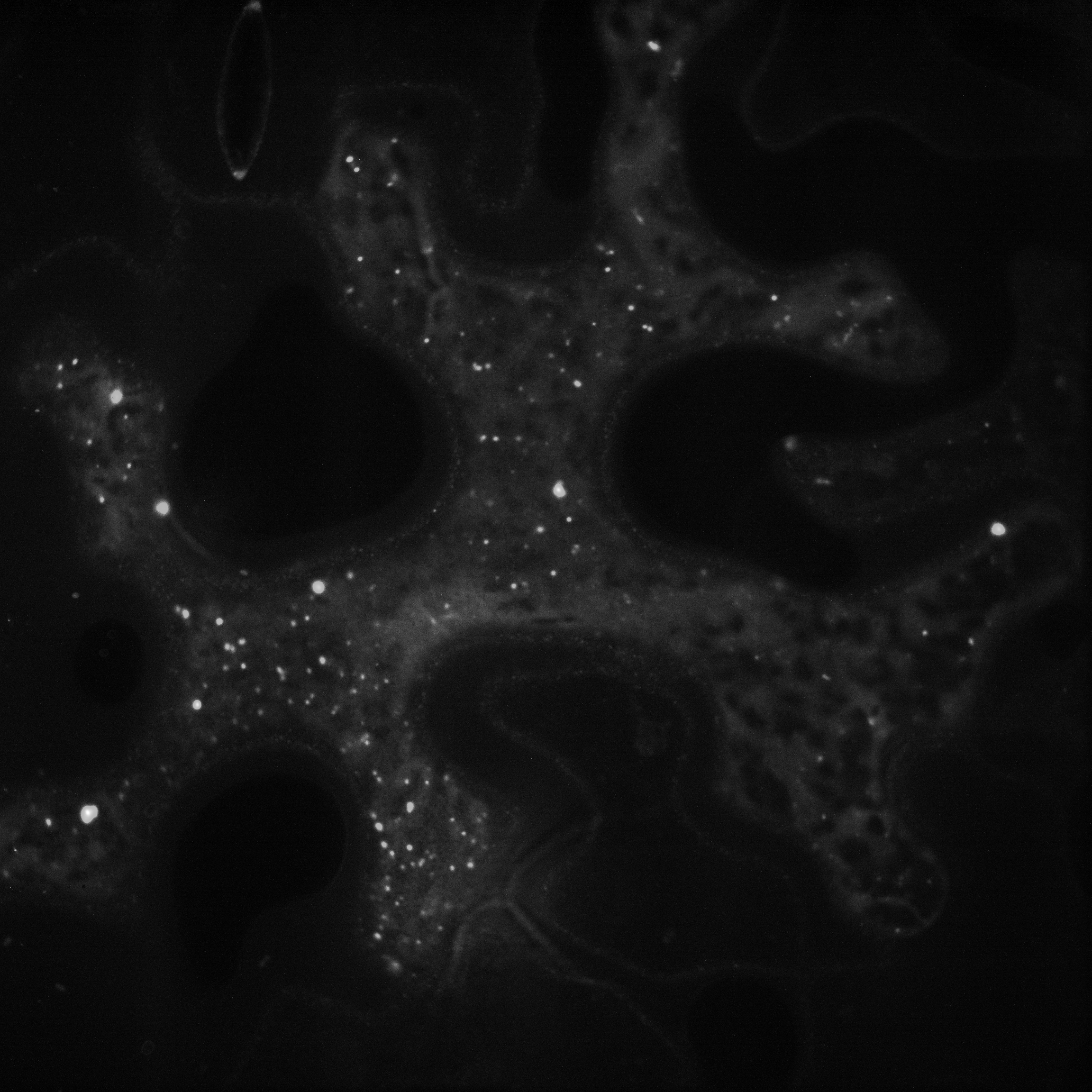

Supplement: Supplementary file 8 — Source data Fig. 4 [file 44318_2024_90_MOESM8_ESM.zip › Figure 4-Source Data Files/4G/Fig 4G-XopAY.tif]

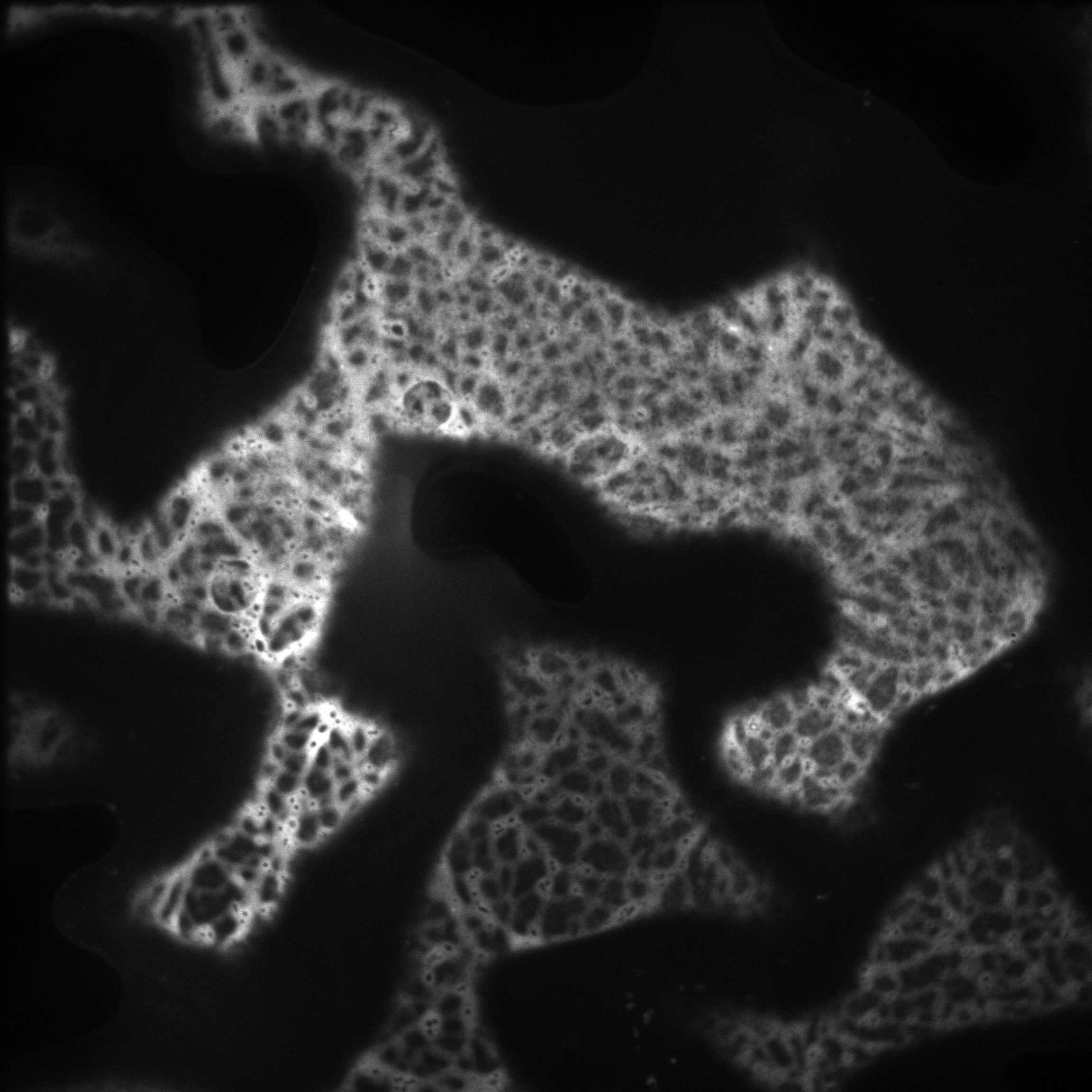

Supplement: Supplementary file 8 — Source data Fig. 4 [file 44318_2024_90_MOESM8_ESM.zip › Figure 4-Source Data Files/4G/Fig 4G-XopAZ.tif]

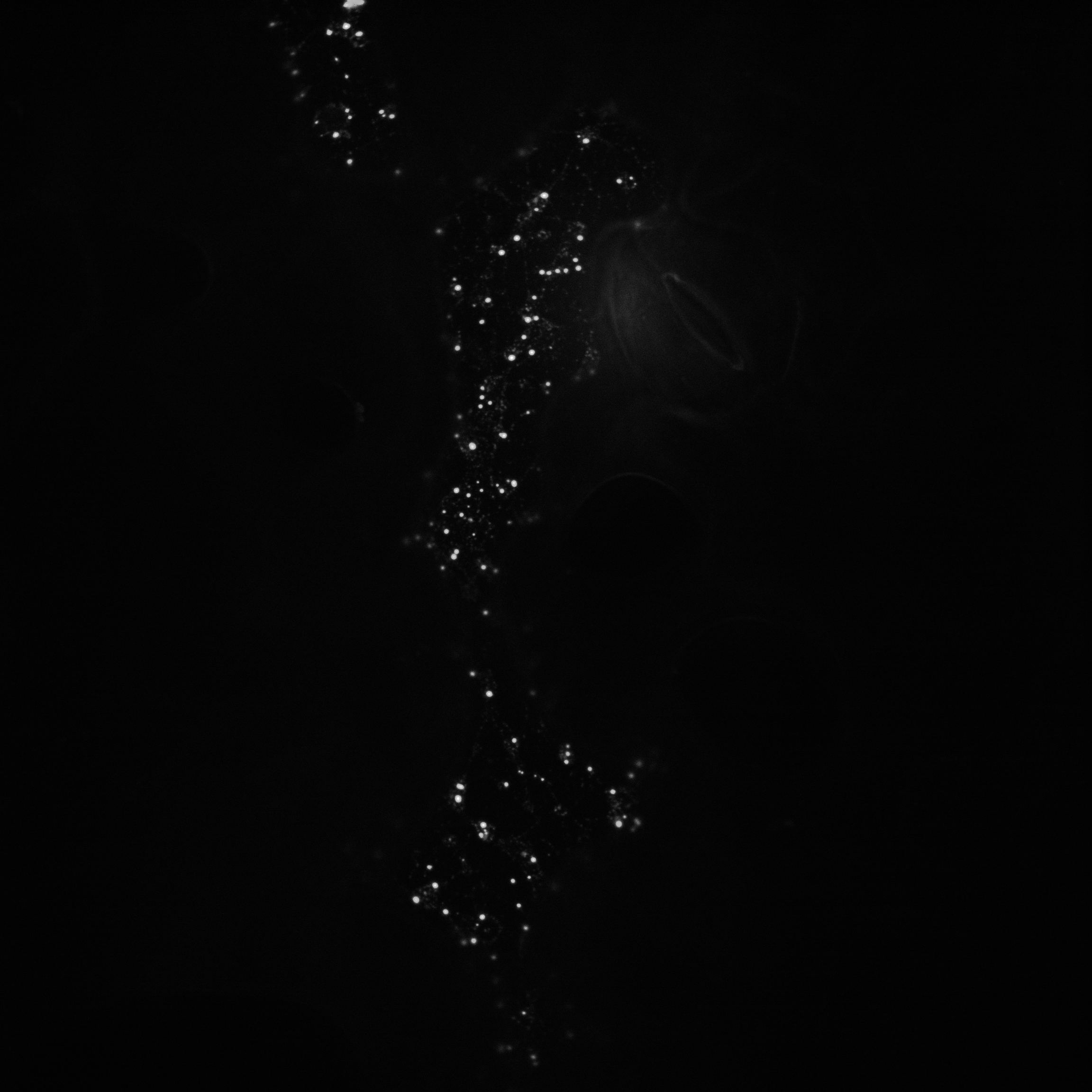

Supplement: Supplementary file 8 — Source data Fig. 4 [file 44318_2024_90_MOESM8_ESM.zip › Figure 4-Source Data Files/4G/Fig 4G-XopD2.tif]

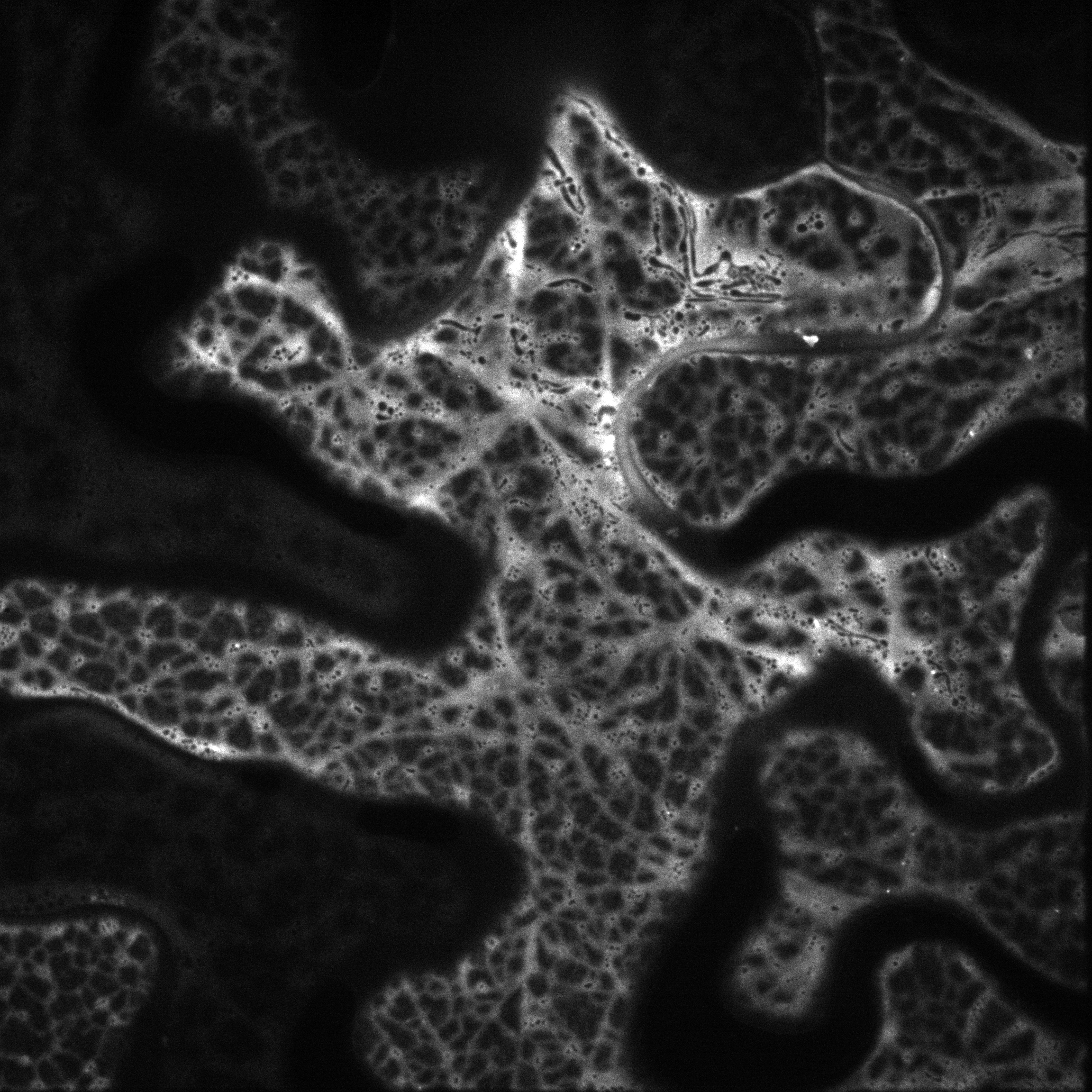

Supplement: Supplementary file 8 — Source data Fig. 4 [file 44318_2024_90_MOESM8_ESM.zip › Figure 4-Source Data Files/4G/Fig 4G-XopQ.tif]

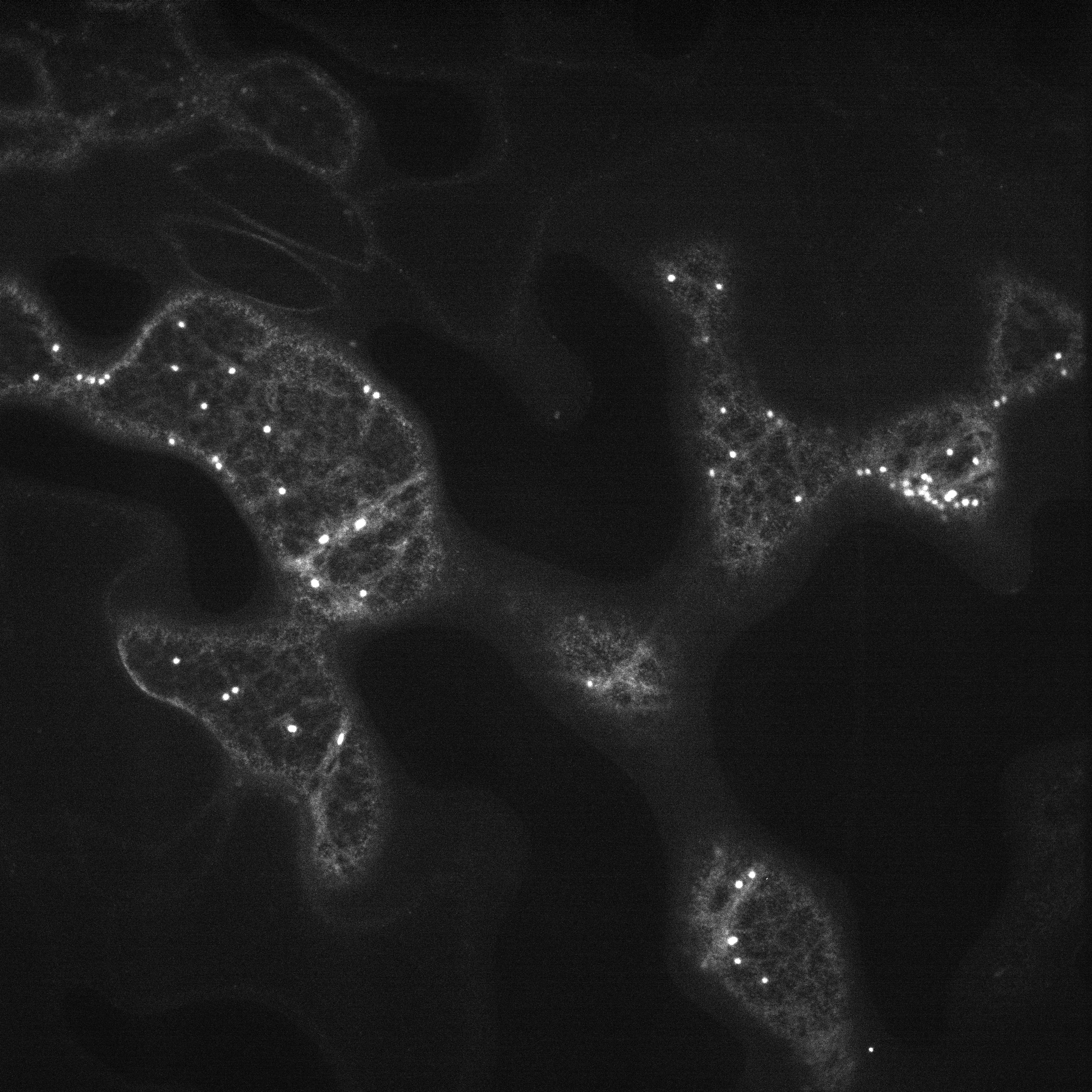

Supplement: Supplementary file 8 — Source data Fig. 4 [file 44318_2024_90_MOESM8_ESM.zip › Figure 4-Source Data Files/4G/Fig 4G-XopX2.tif]

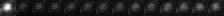

Supplement: Supplementary file 8 — Source data Fig. 4 [file 44318_2024_90_MOESM8_ESM.zip › Figure 4-Source Data Files/4H/Fig 4H-HopA1.tif]

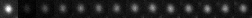

Supplement: Supplementary file 8 — Source data Fig. 4 [file 44318_2024_90_MOESM8_ESM.zip › Figure 4-Source Data Files/4H/Fig 4H-HopS1.tif]
