# Supplementary material for: MolPhase, an advanced prediction algorithm for protein phase separation
Source: EMBO J. 2024 Apr 2;43(9):10. doi: 10.1038/s44318-024-00090-9 (PMC11065880; doi:10.1038/s44318-024-00090-9)
Supplement: Supplementary file 12 — Expanded View Figures [file 44318_2024_90_MOESM12_ESM.pdf]

Expanded View Figures

Figure EV1. Phase separation predictor performance.

(**A**) Workflow for constructing the phase separation predictor. (**B**) 2D vector projection of training datasets prior to one-sided selection undersampling by UMAP. (**C**) Efficacy of seven clustering algorithms on a consistent training set. (**D–G**) Confusion matrices for external dataset predictions by (**D**) DeePhase, (**E**) PSPredictor, (**F**) FuzDrop, and (**G**) PSPer. FuzDrop's cutoff is 0.6, while the others are 0.5, as suggested by their respective studies. PSPer could not process 5 sequences for unspecified reasons, which are excluded from the matrix. Deep green indicates true outcomes, while light green indicates false outcomes. (**H**, **I**) Correlation coefficient of seven features in (**H**) positive training set and (**I**) negative training set. Source data are available online for this figure.

© The Author(s)

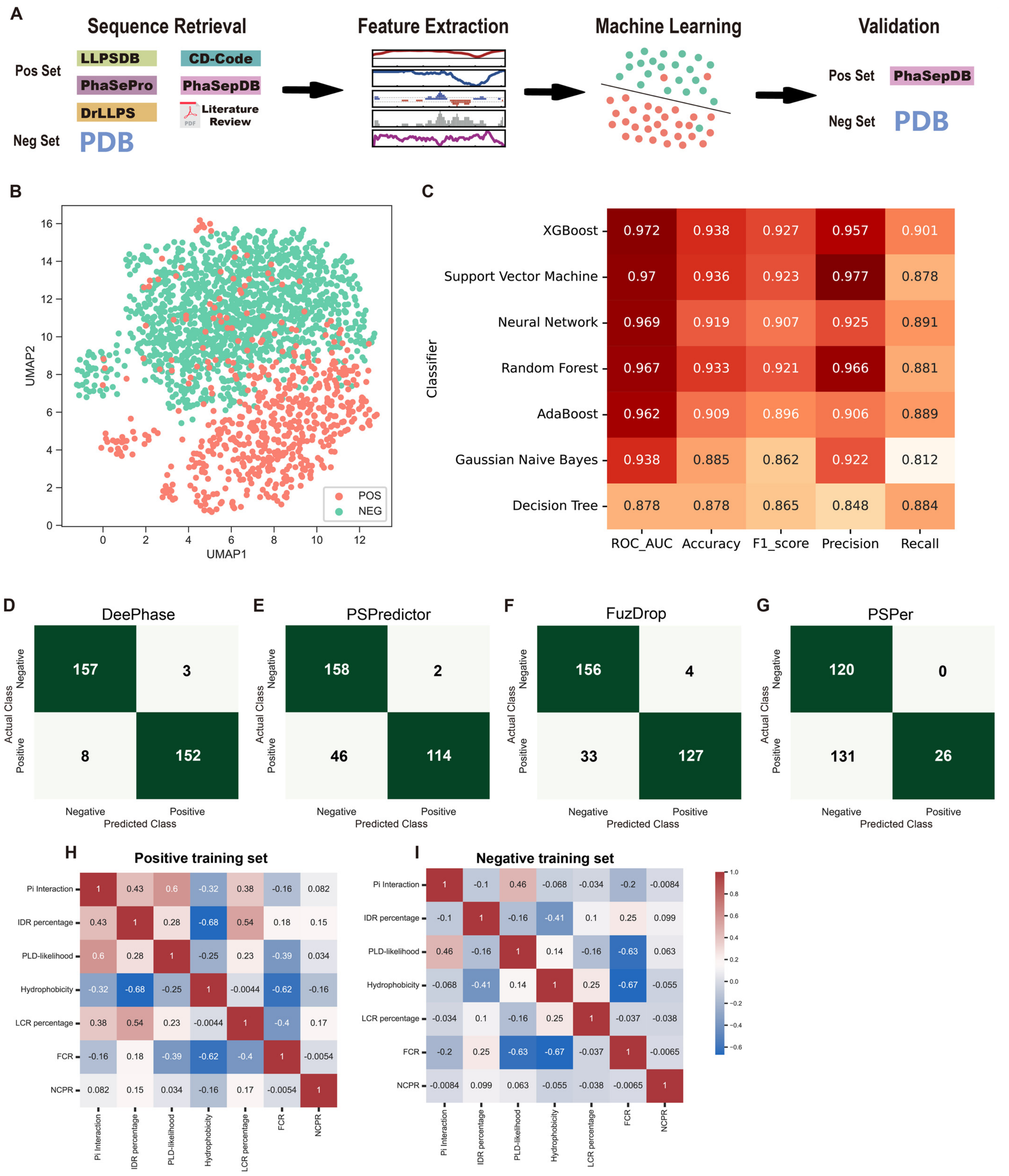
A
Sequence Retrieval
Pos Set
LLPSDB
CD-Code
PhaSePro
PhaSepDB
DrLLPS
Literature Review
Neg Set
PDB
Feature Extraction
Machine Learning
Validation
Pos Set
PhaSepDB
Neg Set
PDB
B
UMAP2
UMAP1
POS
NEG
C
Classifier
XGBoost 0.972 0.938 0.927 0.957 0.901
Support Vector Machine 0.97 0.936 0.923 0.977 0.878
Neural Network 0.969 0.919 0.907 0.925 0.891
Random Forest 0.967 0.933 0.921 0.966 0.881
AdaBoost 0.962 0.909 0.896 0.906 0.889
Gaussian Naive Bayes 0.938 0.885 0.862 0.922 0.812
Decision Tree 0.878 0.878 0.865 0.848 0.884
ROC_AUC Accuracy F1_score Precision Recall
D
DeePhase
157 3
8 152
E
PSPredictor
158 2
46 114
F
FuzDrop
156 4
33 127
G
PSPer
120 0
131 26
Actual Class
Negative
Positive
Predicted Class
H
Positive training set
I
Negative training set
Pi Interaction
IDR percentage
PLD-likelihood
Hydrophobicity
LCR percentage
FCR
NCPR

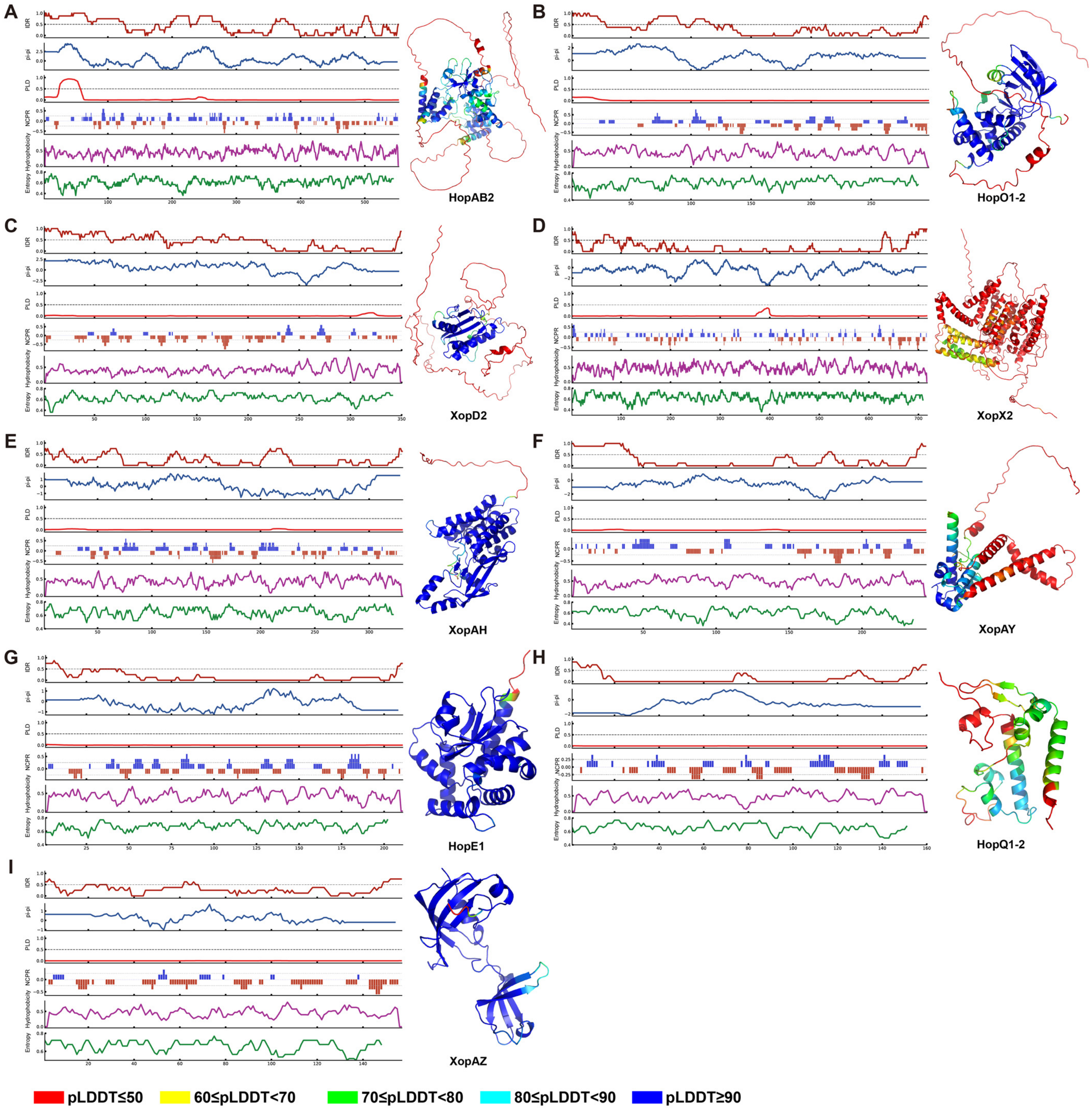

Figure EV2. Effector protein feature extraction and structural prediction.

(**A–I**) Feature extraction and AlphaFold2 structural prediction for four positive candidates: (**A**) HopAB2, (**B**) HopO1-2, (**C**) XopD2, (**D**) XopX2, (**E**) XopAH, (**F**) XopAY, (**G**) HopE1, (**H**) HopQ1-2 and (**I**) XopAZ. Features, from top to bottom, include IDR, pi interaction, prion-like domain likelihood, net charge per residues, hydrophobicity, and Shannon entropy. AlphaFold2-predicted structures are color-coded by predicted local-distance difference test (pLDDT) shown at the bottom of image. Structure image sizes are not to scale. This relates to Fig. 3. Source data are available online for this figure.

 © The Author(s)

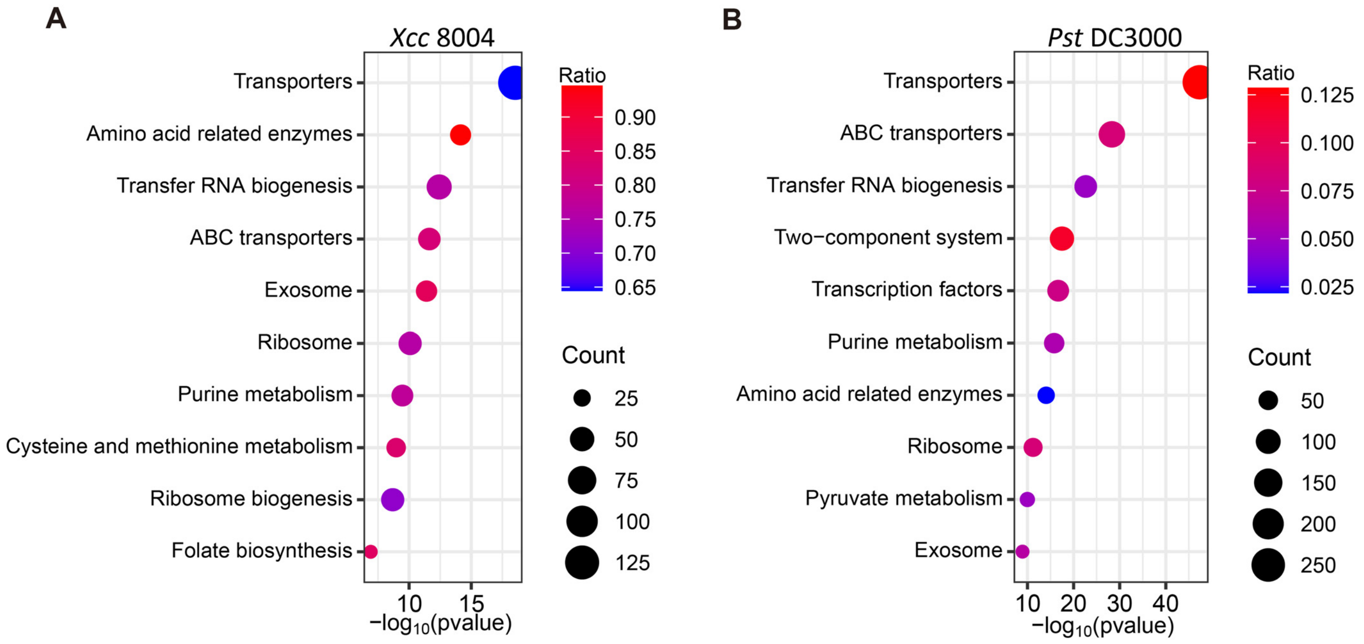

Figure EV3. KEGG analysis for phase separation negative proteins.

(**A**, **B**) KEGG enrichment analysis for proteins with phase separation prediction scores below 0.1 in (**A**) *Xcc* 8004 and (**B**) *Pst* DC3000. The *x* axis shows the $-\log_{10} P$ value for the Fisher exact test, the *y* axis lists enrichment items, the ratio indicates the percentage of the entire pathway enriched, and the count shows the number of enriched items. Source data are available online for this figure.

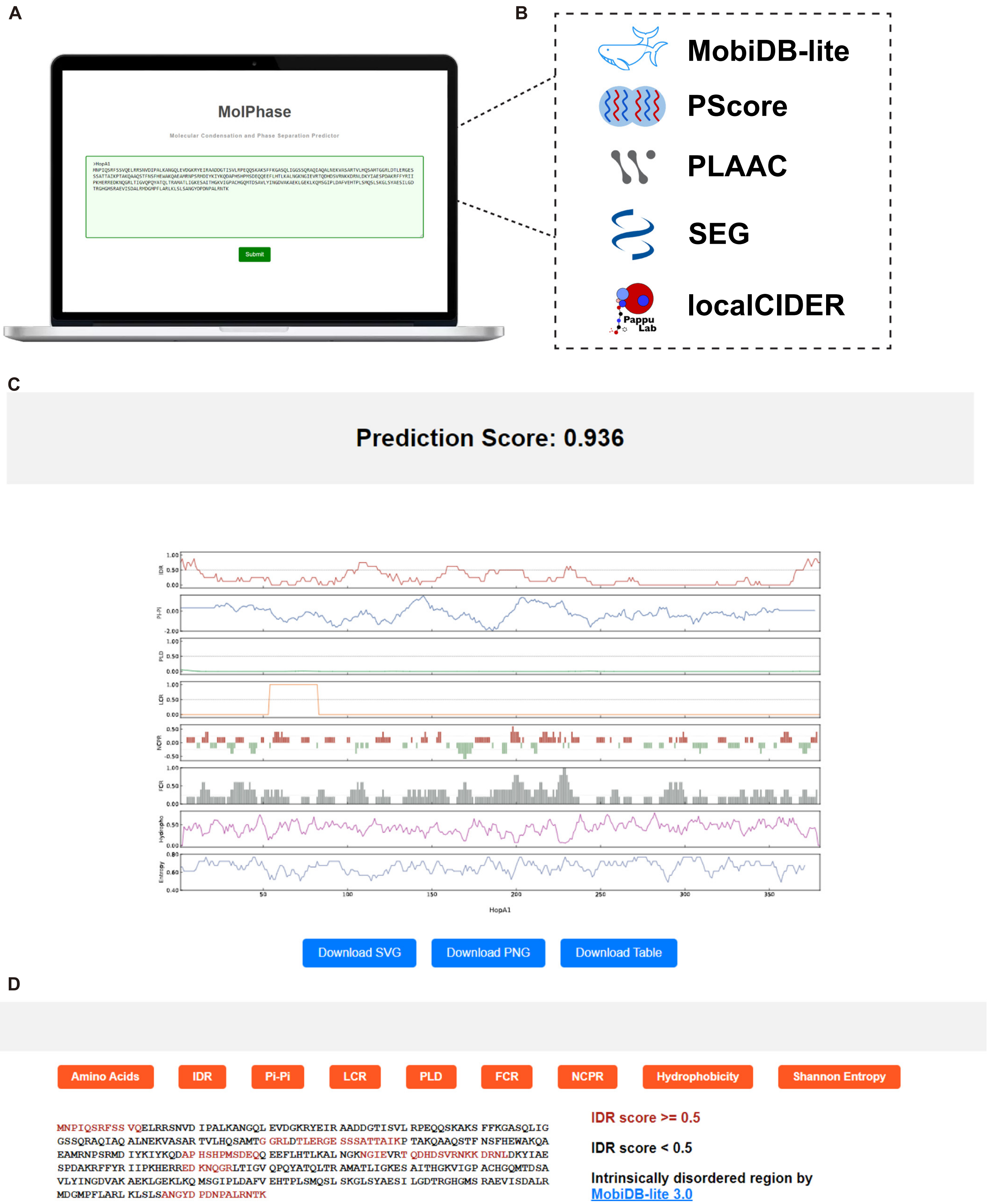
A
MolPhase
Molecular Condensation and Phase Separation Predictor
Submit
B
MobiDB-lite
PScore
PLAAC
SEG
localCIDER
Pappu Lab
C
Prediction Score: 0.936
IDR
Pi-Pi
PLD
LCR
NCPR
FCR
Hydropho
Entropy
HopA1
Download SVG
Download PNG
Download Table
D
Amino Acids
IDR
Pi-Pi
LCR
PLD
FCR
NCPR
Hydrophobicity
Shannon Entropy
MNPIQSRFSS VQELRRSNVD IPALKANGQL EVDGKRYEIR AADDGTISVL RPEQQSKAKS FFKGASQLIG
GSSQRAQIAQ ALNEKVASAR TVLHQSAMTG GRLDTLERGE SSSATTAIKP TAKQAAQSTF NSFHEWAKQA
EAMRNPSRMD IYKIYKQDAP HSHPMSDEQQ EEFLHTLKAL NGKNGIEVRT QDHDSVRNKK DRNLDKYIAE
SPDAKRFFYR IIPKHERRED KNQGRLTIGV QPQYATQLTR AMATLIGKES AITHGKVIGP ACHGQMTDSA
VLYINGDVAK AEKLGEKLKQ MSGIPLDAFV EHTPLSMQSL SKGLSYAESI LGDTRGHGMS RAEVISDALR
MDGMPFLARL KLSLSANGYD PDNPALRNTK
IDR score >= 0.5
IDR score < 0.5
Intrinsically disordered region by MobiDB-lite 3.0

 © The Author(s)

Figure EV4. MolPhase online predictor interface.

(**A**) A screenshot of the MolPhase predictor, displaying the input sequence for the effector HopA1. (**B**) Tools utilized for illustrating features in (**C**). IDR was determined by Mobidb-lite, pi–pi contacts by PScore, LCR by SEG, and PLD by PLAAC. NCPR, FCR, hydrophobicity, and Shannon Entropy were assessed by localCIDER. The displayed score is the phase separation prediction score, ranging from 0 to 1. (**C**) Specific features aiding in predicting potential phase separation proteins, using HopA1 as an example. Features, from top to bottom, are IDR, pi interaction, PLD, LCR, NCPR, FCR, hydrophobicity, and Shannon Entropy. (**D**) Features illustrate in the amino acid sequence view, and feature higher than the threshold score will be highlighted.

© The Author(s)
